# Supplementary material for: Molecular detection of human-derived Jingmenvirus in multiple mosquito species from Yaoundé, Cameroon
Source: Parasit Vectors. 2025 Dec 15;18:505. doi: 10.1186/s13071-025-07111-4 (PMC12723927; doi:10.1186/s13071-025-07111-4)
Supplement: Supplementary file 1 — Additional file 1: Fig. S1 Pairwise comparison of nucleotide sequences generated by sample and segment (S1 to S4), with the human-derived Jingmenvirus (OQ835732-35), the Shuangao virus insect virus 7 (MW314686-89). The upper comparison is the percent identity, and the lower comparison is the number of nucleotide variations. [file 13071_2025_7111_MOESM1_ESM.pdf]

S1	Shuangao Insect virus 7	Human-derived Jingmen virus	Cq_Y_D_22.1	Cq_Y_D_22.2	Cq_Y_R_22.1	Cw_Y_D_22	Cq_Y_R_23
Shuangao Insect virus 7		76.8	96.1	88.1	95.4	82.3	77.2
Human-derived Jingmen virus	676		99.1	99.2	99.7	98.9	98.6
Cq_Y_D_22.1	114	26		99.0	99.9	99.2	99.2
Cq_Y_D_22.2	347	23	29		99.62	99.1	99.0
Cq_Y_R_22.1	133	9	1	11		99.7	99.8
Cw_Y_D_22	518	33	22	25	8		99.2
Cq_Y_R_23	665	40	23	29	7	22	

S3	Shuangao Insect virus 7	Human-derived Jingmen virus	Aa_Y_D_22	Cq_Y_D_22.1	Cq_Y_D_22.2	Cq_Y_R_22.1	Cw_Y_D_22	Ag_G_R_23	Cq_Y_R_23
Shuangao Insect virus 7		79.2	93.9	91.2	88.8	94.5	82.4	98.3	82.5
Human-derived Jingmen virus	552		99.8	99.4	98.4	99.7	99.2	99.9	98.8
Aa_Y_D_22	161	6		99.9	99.8	100.0	99.8	99.9	99.5
Cq_Y_D_22.1	234	15	2		99.3	99.9	99.7	99.9	99.5
Cq_Y_D_22.2	298	41	5	19		99.8	99.1	99.9	98.6
Cq_Y_R_22.1	145	9	0	2	5		99.7	99.9	99.5
Cw_Y_D_22	468	21	5	8	24	9		99.8	99.1
Ag_G_R_23	46	2	1	1	2	2	4		99.8
Cq_Y_R_23	465	32	14	14	36	14	24	5	

S2	Shuangao Insect virus 7	Human-derived Jingmen virus	Aa_Y_D_22	Cq_Y_D_22.1	Cq_Y_D_22.2	Cw_Y_D_22	Cq_Y_R_23
Shuangao Insect virus 7		43.8	83.1	90.1	68.3	68.4	51.0
Human-derived Jingmen virus	907		99.8	99.6	99.6	99.7	99.4
Aa_Y_D_22	272	3		99.9	99.9	99.9	99.8
Cq_Y_D_22.1	159	6	1		99.7	99.7	99.4
Cq_Y_D_22.2	511	7	2	5		99.7	99.4
Cw_Y_D_22	510	5	1	5	5		99.7
Cq_Y_R_23	792	10	3	9	9	5	

S4	Shuangao Insect virus 7	Human-derived Jingmen virus	Aa_Y_D_22	Cq_Y_D_22.1	Cq_Y_D_22.2	Cq_Y_R_22.1	Cq_Y_R_22.2	Cw_Y_D_22	Cq_Y_R_23
Shuangao Insect virus 7		74.7	96.0	97.3	89.5	97.8	98.8	84.8	78.3
Human-derived Jingmen virus	634		99.7	99.6	98.0	99.7	99.8	99.2	98.9
Aa_Y_D_22	100	8		100.0	99.9	100.0	99.9	100.0	99.8
Cq_Y_D_22.1	67	9	0		100.0	100.0	100.0	99.9	99.7
Cq_Y_D_22.2	262	51	1	0		99.8	99.9	98.8	98.1
Cq_Y_R_22.1	54	7	0	0	4		100.0	99.9	99.9
Cq_Y_R_22.2	31	6	1	0	2	0		99.9	99.9
Cw_Y_D_22	382	21	0	3	30	1	1		99.3
Cq_Y_R_23	543	28	6	7	47	2	2	18	

The upper comparison is the percent identity, and the lower comparison is the number of nucleotide variations
