# Supplementary figures and images for: Molecular detection of human-derived Jingmenvirus in multiple mosquito species from Yaoundé, Cameroon
Source: Parasit Vectors. 2025 Dec 15;18:505. doi: 10.1186/s13071-025-07111-4 (PMC12723927; doi:10.1186/s13071-025-07111-4)

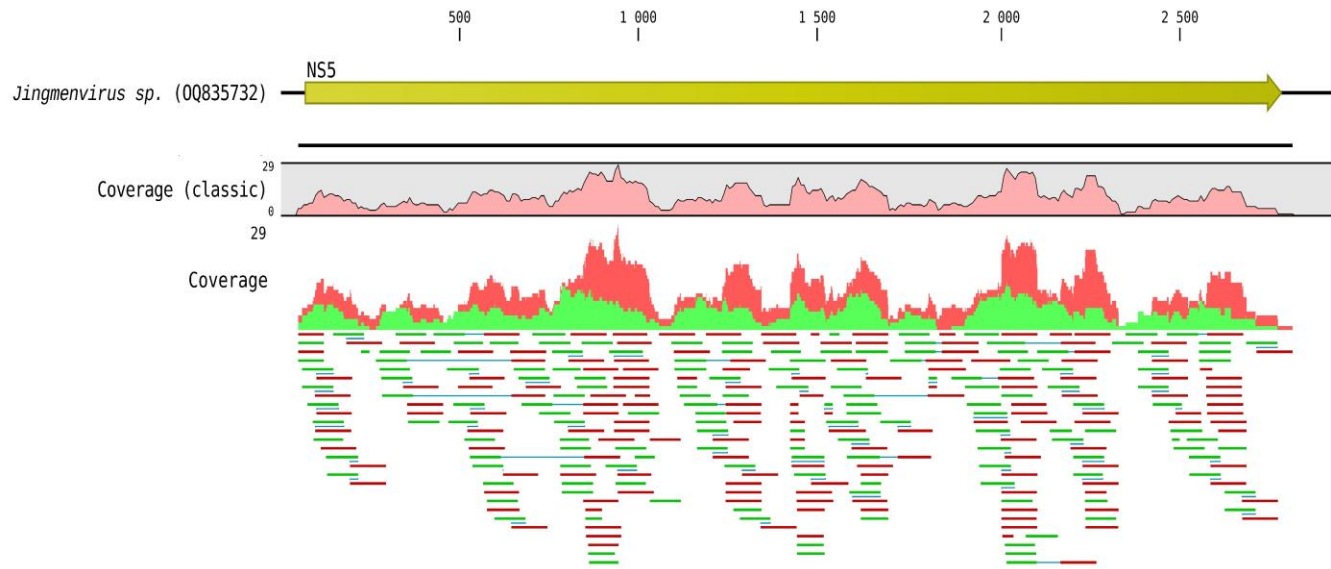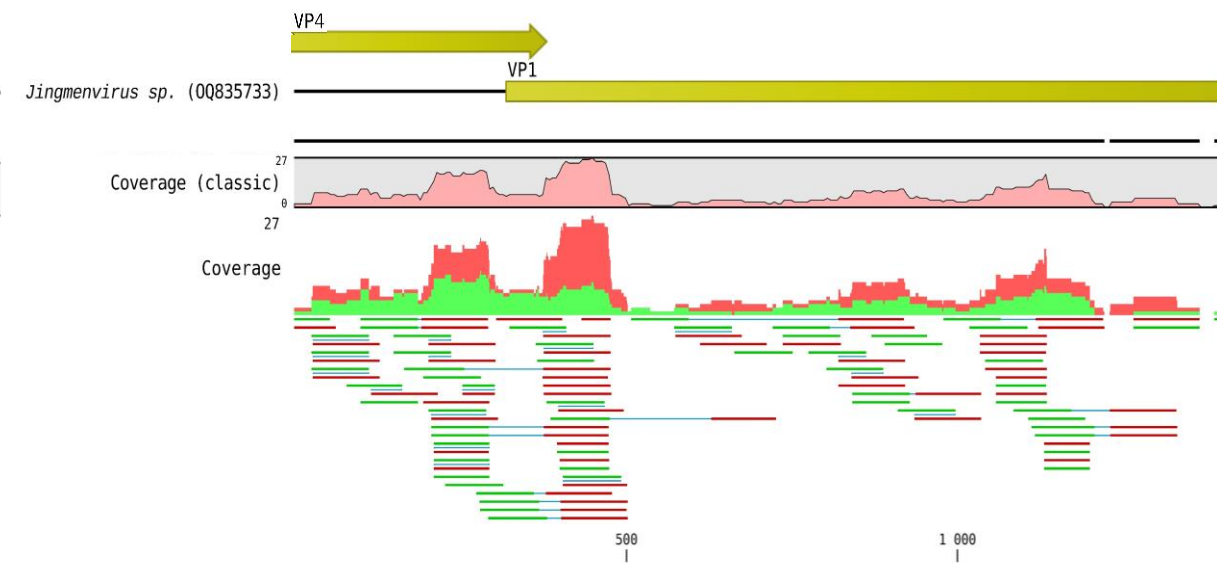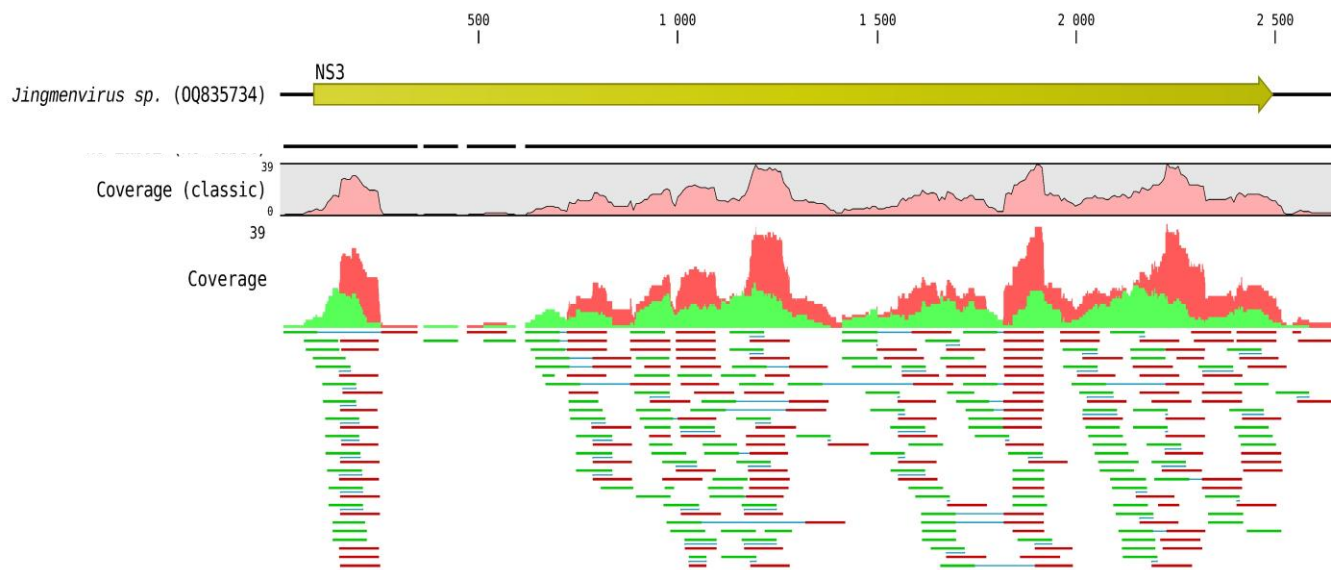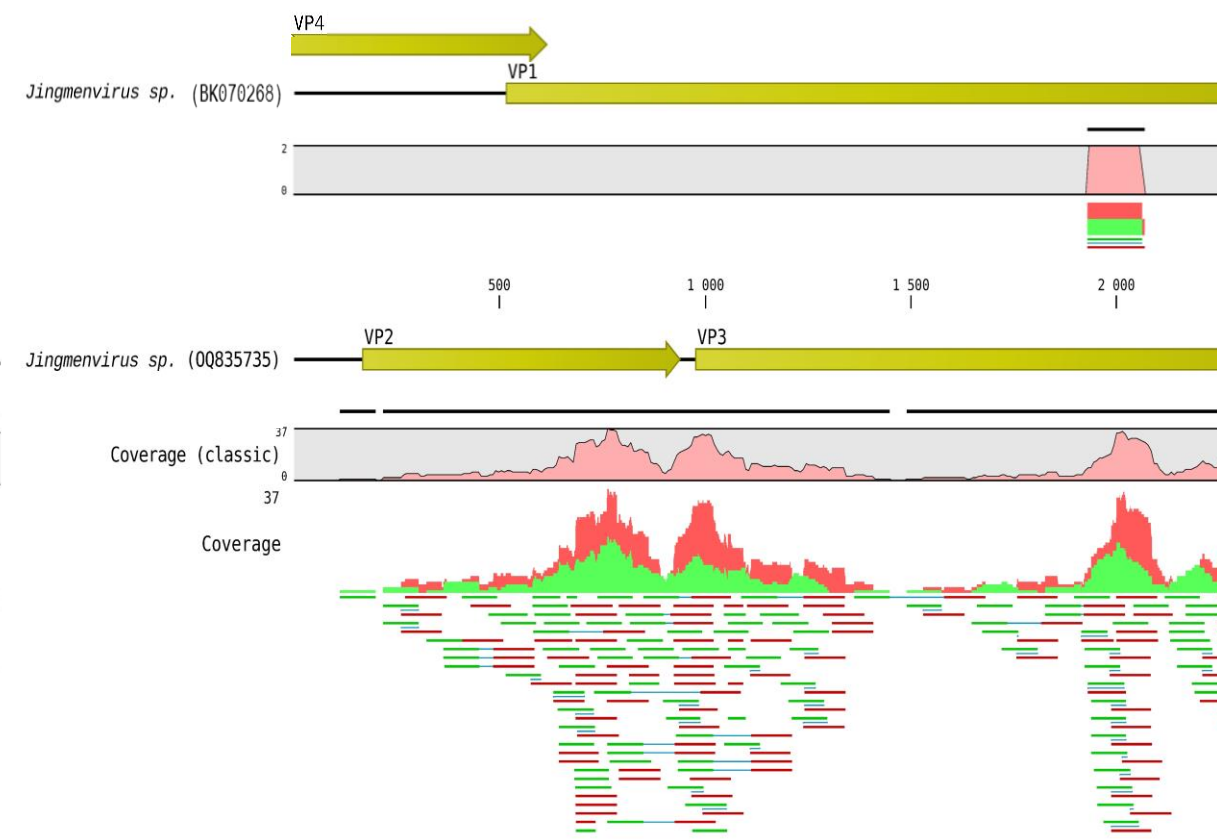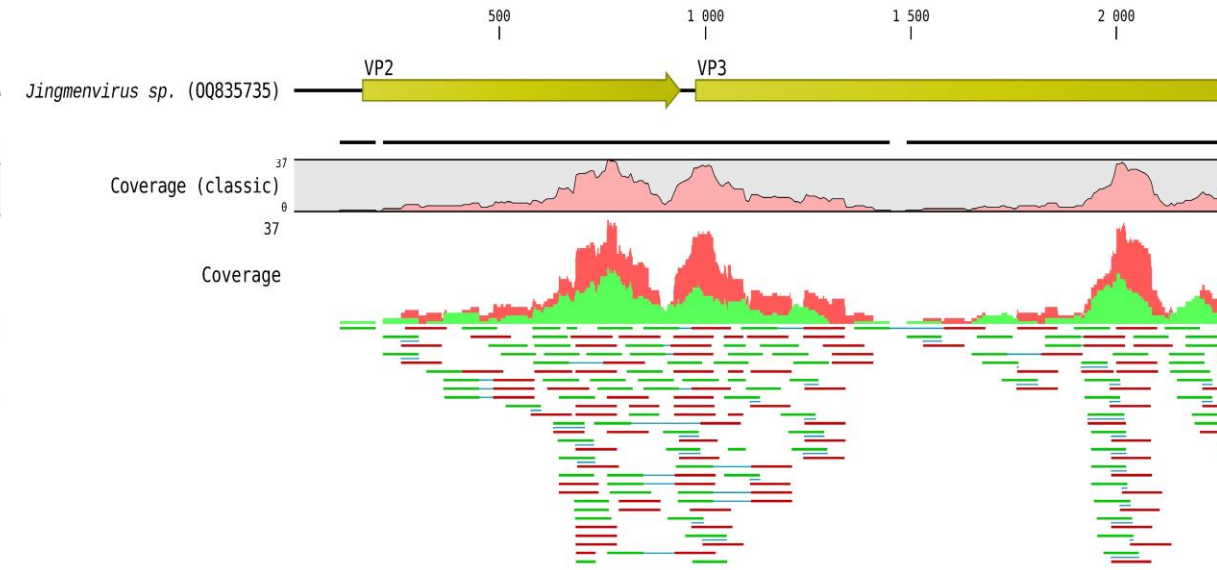

Supplement: Supplementary file 2 — Additional file 2: Fig. S2 Mapping of all positive samples reads on the HdJV genomic segments OQ835735, OQ835734, OQ835733, OQ835732, and BK070268. The lines below the sequences correspond to the different reads that have mapped to the reference. Green indicates that the single read is in the same direction as the reference, red indicates single reads in the antisense direction, and blue indicates paired reads. [file 13071_2025_7111_MOESM2_ESM.pdf]
