# Supplementary material for: Molecular detection of human-derived Jingmenvirus in multiple mosquito species from Yaoundé, Cameroon
Source: Parasit Vectors. 2025 Dec 15;18:505. doi: 10.1186/s13071-025-07111-4 (PMC12723927; doi:10.1186/s13071-025-07111-4)
Supplement: Supplementary file 3 — Additional file 3: Table S1. Name, accession numbers of the four consensus sequences generated from our different positive samples MaJV (Mosquitoes associated Jingmenvirus), and comparison with the human-derived Jingmenvirus (HdJV) sequences of reference. [file 13071_2025_7111_MOESM3_ESM.docx]

Additional file 3: Table S1. Name, accession numbers of the four consensus sequences generated from our different positive samples MaJV (Mosquitoes associated Jingmenvirus), and comparison with the human-derived Jingmenvirus (HdJV) sequences of reference.

| Name | Accession Number Genbank | % coverage of the reference | % identity | Reference Accession Number Genbank |
| --- | --- | --- | --- | --- |
| Jingmenvirus sp. Cameroon Mosquitoes MaJV1 segment1 | PV953369 | 93.6 | 98.9 | OQ835732 |
| Jingmenvirus sp. Cameroon Mosquitoes MaJV2 segment2 | PV953370 | 98.1 | 97.8 | OQ835733 |
| Jingmenvirus sp. Cameroon Mosquitoes MaJV3 segment3 | PV953371 | 96.7 | 96.6 | OQ835734 |
| Jingmenvirus sp. Cameroon Mosquitoes MaJV4 segment4 | PV953372 | 93.4 | 96.4 | OQ835735 |
